# Levosimendan: efficacy and safety in pediatric heart failure treatment

**DOI:** 10.1590/1806-9282.20240257

**Published:** 2024-07-19

**Authors:** Mehmet Akif Dündar, Mustafa Yılmaz, Mustafa Argun

**Affiliations:** 1 Health Sciences University, Kayseri City Training and Research Hospital, Department of Pediatrics, Division of Pediatric Intensive Care – Kayseri, Turkey.; 2 Health Sciences University, Kayseri City Training and Research Hospital, Department of Pediatric Cardiovascular Surgery – Kayseri, Turkey.; 3 Health Sciences University, Kayseri Medical Faculty, Department of Pediatrics, Division of Pediatric Cardiology – Kayseri, Turkey.

**Keywords:** Cardiac inotropism, Heart Failure, Levosimendan, Low cardiac output syndrome, Myocarditis, Pediatrics

## Abstract

**OBJECTIVE:**

The objective of this study was to assess the effectiveness and safety of levosimendan as an alternative treatment for pediatric patients with decompensated heart failure unresponsive to conventional inotropes and to emphasize its role in enhancing cardiovascular stability.

**METHODS:**

A total of 15 pediatric patients with decompensated heart failure, stemming from acute fulminant myocarditis (53.3%) and post-congenital heart disease surgery complications (46.7%), received levosimendan. The evaluation focused on adverse effects, respiratory support requirements, and concurrent inotropic medication use during levosimendan treatment. Key cardiovascular parameters were assessed at 0, 6, 12, and 24 h post-levosimendan infusion.

**RESULTS:**

Levosimendan administration significantly improved key cardiovascular metrics. Left ventricular ejection fraction increased notably from 45±14.8% to 58±15.6% at 24 h (p<0.001). Systolic and diastolic blood pressures rose significantly, with systolic increasing from 79 (68–90) to 98 (89–109) mmHg and diastolic from 47 (40–57) to 66 (54–76) mmHg by 24 h (p<0.001). Heart rate decreased from 162 (111–175) to 132 (99–148) bpm (p=0.02), and lactate levels significantly decreased from 4.15 (2.3–6.5) to 1.85 (0.8–2.6) mmol/L within 6 h (p<0.001).

**CONCLUSION:**

Levosimendan demonstrates its significance in managing pediatric heart failure, indicating its safety and potential to enhance cardiac outcomes by reducing reliance on traditional inotropes.

## INTRODUCTION

Heart failure (HF) is a complex clinical syndrome characterized by structural or functional cardiac abnormalities that impair the heart's ability to fill with or eject blood. This deficiency leads to inadequate tissue perfusion and an inability to meet the body's metabolic demands^1^. Acute HF is distinguished by symptoms of congestion, reduced blood flow, tachycardia, and hypotension, often resulting from rapid changes in cardiac structure or function over minutes to hours^2^. In pediatric populations, HF's etiology significantly differs from adults, predominantly due to congenital heart disease, postoperative reperfusion injury, and severe myocarditis^3^.

Low cardiac output syndrome (LCOS) describes a state of reduced cardiac output resulting from transient myocardial dysfunction, commonly observed in severe sepsis, myocarditis, and various cardiomyopathies, and as a significant complication after cardiac surgery^4^. LCOS is identified by a constellation of laboratory and clinical signs, including elevated blood lactate levels, low central venous oxygen saturation, decreased urine output, reduced left ventricular ejection fraction (LVEF), and an increased need for inotropic support^5^.

The ongoing research for an optimal inotropic agent to treat acute decompensated HF highlights the limitations of current therapies in reducing symptoms and morbidity. With its unique mechanism of enhancing myocardial contractility without increasing myocardial oxygen demand, levosimendan has shown promise in cases of decompensated HF refractory to standard therapy^6^. By sensitizing myocardial cells to calcium at the systolic phase while preserving diastolic function and preventing cellular damage through controlling intracellular calcium influx, levosimendan improves ventricular function^7^.

Although studies have shown efficacy in improving cardiac function in the pediatric intensive care unit (PICU), in the treatment of severe HF, and in the management of acute fulminant myocarditis and postoperative cardiac conditions, there are no established guidelines^8^. Conventional inotropic therapies are sometimes inadequate despite high doses. New treatment modalities and guidelines are needed for decompensated HF in children. At this stage, levosimendan is a very promising agent that may replace some of the agents in the treatment modalities. This study evaluates the efficacy and safety of levosimendan in pediatric patients with LCOS and decompensated HF following cardiac surgery or acute myocarditis. The therapeutic benefit of levosimendan in improving cardiac output and overall cardiovascular stability in this vulnerable population will be assessed.

## METHODS

### Study design and population

A retrospective analysis was conducted, examining clinical records of pediatric patients administered levosimendan from May 1, 2017, to July 1, 2019, in the PICU at Health Sciences University, Kayseri City Hospital. Approval was obtained from the Ethics Committee of Erciyes University for the use of patient data, and informed consent was secured from legal guardians.

A total of 15 pediatric individuals who were diagnosed with decompensated HF, post-acute myocarditis, or following cardiac surgery and were treated with levosimendan were included in this study. Exclusion criteria involved patients with chronic comorbidities.

### Data collection method

Patient records were retrospectively reviewed for diagnostic specifics, etiology of HF, prior medication use, results of physical examinations, laboratory data, arterial blood gas and lactate levels, levosimendan dosage, and vital signs (including blood pressure and heart rate) during treatment, as well as urine output.

### Therapeutic approach

Before levosimendan administration, epinephrine and milrinone were used as standard inotropic therapies. Levosimendan was introduced in cases of persistent low cardiac output despite high-dose inotropic therapy, characterized by tachycardia, elevated lactate levels, reduced ejection fraction, and low systolic pressure. Administration began 48–96 h after acute HF onset, with a loading dose of 12 pg/kg over 1 h, followed by a continuous infusion of 0.1 pg/kg/min for 24 h, unless adverse effects necessitated dosage modification. Treatment was carried out in the PICU, with continuous arterial monitoring and rhythm surveillance for real-time tracking of blood pressure and heart rate.

### Vasoactive inotropic score calculation

The VIS was calculated using the following equation: [dopamine(μg/kg/min)]+[dobutamine(μg/kg/min)]+[100×epinephrine(μg/kg/min)]+[10×milrinone(μg/kg/min)]+[10,000×vasopressin(U/kg/min)]+[100×norepinephrine(μg/kg/min)] . Data were collected at baseline (T0) and at 6, 12, and 24 h (T6, T12, and T24) following the initiation of the levosimendan infusion, as extracted from the ICU database.

### Statistical analyses

Data were analyzed using SPSS for Windows (Version 22.0) and Sigma Stat (Version 3.1). The Shapiro-Wilk test was applied to determine the distributional characteristics of all variables. Parameters with normal distribution were reported as mean±SD, while those with non-normal distribution were presented as median (interquartile range: 25th–75th percentile). For intra-group comparisons, parametric variables were analyzed using the paired-sample t-test, and non-parametric variables were assessed via the Wilcoxon test. The Friedman test was employed for intergroup comparisons of non-parametric data, including hemodynamic and blood gas values and echocardiographic parameters. A p-value of <0.05 was considered statistically significant.

## RESULTS

### Patient demographics and heart failure etiology

The study encompassed 15 pediatric patients treated with levosimendan for HF. The detailed characteristics of HF are presented in Table 1. Acute fulminant myocarditis was identified as the primary etiology in 53.7% (8 patients), while the remaining 46.7% (7 patients) had postoperative complications from congenital heart surgeries. The surgeries included ventricular septal defect repair in 33.3% (5 patients), atrioventricular septal defect repair in 6.7% (1 patient), and tetralogy of Fallot repair in 6.7% (1 patient). There was one fatality on the seventh postoperative day in a patient with tetralogy of Fallot, and all other patients survived.

**Table 1:** Patient characteristics and etiology in the administration of levosimendan.

Patients (numbers)		15
Age, months		20 (2–156 min–max.)
Length, cm		76 (54–158 min–max.)
Weight, kg		11.2 (4.2–62 min–max.)
Male/female, n		7/8
Etiology of heart failure		
	Acute fulminant myocarditis	8 (53.4%)
	Postoperative cardiac surgery	7 (46.7%)

### Respiratory support and inotropic therapy during levosimendan treatment

Data on respiratory support and inotropic therapies during levosimendan administration are detailed in Table 2. Mechanical ventilation was required for 66.7% (10 patients), while high-flow oxygen was provided to 33.3% (5 patients).

**Table 2:** Overview of respiratory support and concomitant inotropic medications during levosimendan administration.

Patients (n=15)		n (%)
Respiratory support therapy		
	Mechanically ventilated	10 (66.7%)
	High flow oxygen	5 (33.3%)
Concomitant inotropes		
	Epinephrine + milrinone	10 (66.6%)
	Epinephrine + milrinone + dopamine	2 (13.3%)
	Epinephrine + milrinone + norepinephrine	3 (20%)

Concomitant inotropic therapy included a combination of epinephrine and milrinone in 66.6% (10 patients), epinephrine, milrinone, and dopamine in 13.3% (2 patients), and epinephrine, milrinone, and norepinephrine in 20% (3 patients).

### Analysis of hemodynamic, blood gas, and echocardiographic parameters

Significant improvements were observed in several parameters following levosimendan infusion. LVEF increased from 45±14.8% at baseline to 58±15.6% at 24 h (p<0.001, Table 3). Systolic pressure showed significant increases at 12 h [91 (83–101) mmHg] and 24 h [98 (89–109) mmHg] compared with baseline [79 (68–90) mmHg] (p<0.001). Diastolic pressure also increased significantly at 24 h [66 (54–76) mmHg] versus baseline [47 (40–57) mmHg] (p<0.001). Heart rate decreased from 162 (111–175) at baseline to 132 (99–148) at 24 h (p=0.02). Lactate levels decreased significantly from 6 h [1.85 (0.8–2.6) mmol/L] compared with baseline [4.15 (2.3–6.5) mmol/L] (p<0.001, Table 3). No significant changes were observed in partial arterial carbon dioxide pressure (PaCO_2_), urine output, and vasoactive inotropic score (p=0.39, p=0.09, and p=0.11, respectively). A summary of hemodynamic parameters after a 24-h levosimendan infusion is presented in Table 3.

**Table 3:** Changes in hemodynamic, blood gas, and echocardiographic parameters during 24-h levosimendan infusion.

	Before 0 h (n=15)	6 h (n=15)	12 h (n=15)	24 h (n=15)	p
*Heart rate (bpm)*	162 (111–175)	139 (114–163)	127 (104–151)	132 (99–148)^a^	**0.02**
*Systolic pressure (mmHg)*	79 (68–90)	89 (82–100)	91 (83–101)^b^	98 (89–109)^a^	**<0.001**
*Diastolic pressure (mmHg)*	47 (40–57)	55 (47–65)	61 (53–67)	66 (54–76)^a^	**<0.001**
*pH*	7.36 (7.29–7.44)	7.4 (7.35–7.43)	7.46 (7.39–7.50)^b^	7.45 (7.38–7.48)	**0.04**
*Lactate (mmol/L)*	4.15 (2.3–6.5)	1.85 (0.8–2.6)^c^	1.0 (0.8–1.85)	1.04 (0.8–1.45)	**<0.001**
*pCO*_2_ *(mmHg)*	44 (29–49)	45 (36–48)	39 (33–42)	40 (35–46)	0.39
*Urine output (mL/kg/h)*	3.2 (1.8–5.07)	_	_	4.2 (2.7–5.2)	**0.09**
*LV ejection fraction, %*	45±14.8	_	_	58±15.6^a^	**<0.001**
*Vasoactive inotropic score*	60 (40–90)			55 (40–80)	0.11

Data are presented as mean ± SD or as median (25th–75th percentile), as appropriate. Notations a, b, and c indicate statistically significant differences: (a) between baseline and 24 h, (b) between baseline and 12 h, and (c) between baseline and 6 h, respectively. Statistically significant p-values are highlighted in bold.

## DISCUSSION

The results of this investigation have highlighted the efficacy of levosimendan in improving cardiac function in pediatric patients with HF, particularly following acute fulminant myocarditis and post-operative congenital heart disease scenarios. Initiated with the aim of exploring the role of levosimendan in a pediatric setting, this study has supported the hypothesis that levosimendan can significantly improve heart rate, systolic and diastolic pressure, pH, and lactate levels, thereby improving cardiac function. Such findings reflect the growing interest in identifying and optimizing treatment strategies that can improve clinical outcomes in this vulnerable population, positioning levosimendan as a potential linchpin in the management of pediatric HF.

In reviewing the strengths of this study, it is important to highlight the novel insights it provides into the use of levosimendan in children. Despite well-documented efficacy in adult patients, pediatric-specific evidence remains relatively scarce. The study fills this gap by demonstrating the safety and therapeutic benefits of levosimendan in children, a cohort previously underrepresented in HF research. Notably, levosimendan was administered predominantly in cases where conventional inotropic support failed to maintain stable hemodynamics, highlighting its utility in challenging clinical situations.

The safety and efficacy of levosimendan for HF treatment have been well-established in adults, prompting its consideration for pediatric applications^9^. The adult-centric evidence base contrasts with the relatively sparse pediatric data, particularly for children with specific heart conditions such as cardiomyopathy or those experiencing LCOS when alternative inotropic treatments prove inadequate. Levosimendan's pediatric use shines in its capacity to support children through various challenging conditions, including post-cardiac surgery, cardiomyopathy, and HF, as demonstrated in a study with 27 children^10^. Our research adds to this growing evidence by highlighting levosimendan's effectiveness in pediatric patients afflicted with acute fulminant myocarditis and those facing postoperative complications following surgeries for congenital heart defects such as ventricular septal defects, atrioventricular septal defects, and tetralogy of Fallot, who exhibited low cardiac output in spite of intensive inotropic therapy.

The acknowledgment of levosimendan's potential to defer the need for mechanical assistive devices in pediatric cases of cardiomyopathy was documented in a survey where 89% of responding clinicians reported positive outcomes^11^. This aligns with our observations, showing levosimendan as a crucial stabilizer of hemodynamics, circumventing the immediate need for mechanical interventions. Furthermore, a meta-analysis encompassing 1,036 patients, both pediatric and adult, underscored levosimendan's ability to effectively lower serum lactate levels and bolster cardiac function^12^, mirroring our findings among children with acute fulminant myocarditis and postoperative congenital heart diseases.

In detailing the effects of levosimendan post-congenital heart surgery, one study noted its advantages in 64 children, including reduced lactate levels, improved cardiac output, and maintained vasoactive inotropic scores^13^. Another piece of research found diminished inotropic support requirements, better ejection fraction, and decreased lactate levels following levosimendan treatment in 15 children, specifically addressing LCOS following cardiac surgeries^14^. These outcomes resonate with our study, which observed significant improvements in cardiac output parameters and lactate levels, pointing to enhanced myocardial contractility and tissue oxygenation in children with diagnoses of acute fulminant myocarditis and post-surgical complications from congenital heart repairs.

Contrasting insights emerge on renal function and urine output. Some studies suggest renal benefits from levosimendan^15^, yet our investigation did not reveal notable changes in urine output, implying that the synergistic effect of inotropes and inodilators, like milrinone, was likely instrumental in preserving renal perfusion across our patient group, particularly those recovering from congenital heart surgeries.

The respiratory advantages of levosimendan, particularly in adult cohorts with challenges in weaning from mechanical ventilation, have been documented^16^. Our findings, showing beneficial impacts on pH and lactate levels without significant alterations in blood carbon dioxide levels, hint at a targeted improvement in metabolic efficiency and potentially respiratory muscle functionality, especially relevant for children with acute fulminant myocarditis and those recovering from surgical interventions for congenital heart defects.

Addressing the limitations of our study is critical to contextualizing its contributions. The retrospective design and modest sample size of 15 patients may limit the extrapolation of our findings to the broader pediatric population. Furthermore, the etiological diversity within our cohort, ranging from acute fulminant myocarditis to post-cardiac surgery conditions, requires a cautious interpretation of the results. Such heterogeneity highlights the complexity of pediatric HF and underscores the need for further research tailored to different patient subgroups.

Considering the wider implications of levosimendan use, it is clear that this study adds to the existing literature by providing pediatric-specific data, thereby filling a critical gap. As demonstrated in our cohort, the safety and efficacy of levosimendan suggest its potential to reduce reliance on traditional inotropes, potentially mitigating associated risks and improving patient outcomes. This finding is invaluable to clinicians navigating the complex landscape of pediatric HF management and offers a glimmer of hope for improved therapeutic strategies.

In conclusion, this study represents a significant advance in our understanding of the role of levosimendan in the management of pediatric HF. By demonstrating its beneficial effects on key cardiac parameters and its safety profile, the study not only adds to the growing body of evidence supporting the use of levosimendan in children but also highlights the need for further research. Future studies, ideally with larger sample sizes and prospective designs, are essential to fully delineate the efficacy of levosimendan and optimize its use in pediatric HF, ultimately aiming to improve the quality of care and outcomes for this vulnerable patient population.

## ETHICAL ASPECTS

This study was conducted in accordance with the ethical standards outlined by the responsible committee for human experimentation and in compliance with the Helsinki Declaration. Institutional Ethical Committee approval was obtained, and informed consent was secured from all participants involved in the research.
